# Sugar substitutes and taste enhancers need more science, sensitivity- and allergy-guided labeling

**DOI:** 10.1038/s41538-023-00240-z

**Published:** 2023-12-09

**Authors:** D. A. Steindler

**Affiliations:** The Eshelman Institute for Innovation, The University of North Carolina at Chapel Hill and Steindler Consulting, Boston, MA USA

**Keywords:** Risk factors, Diseases

## Abstract

There is new attention to food safety resulting from the second White House Conference on Hunger, Nutrition, and Health, as well as new advisories from the World Health Organization calling for more research on sugar substitutes because of possible cancer risks. Together they point to a need for rethinking how we study sugar substitutes and taste enhancers as potentially contributing to adverse health changes. In addition to the need for more research on sweeteners and taste enhancers, including the use of sensitive bioassays, and epidemiological and human clinical trial studies, there should be a call for better truth in labeling, especially including single names for such dietary elements that would afford easier recognition and potential avoidance by those with sensitivities and allergies.

On the heels of the second, some 50 years after the first, “White House Conference on Hunger, Nutrition and Health”, along with the “Biden-Harris Administration National Strategy on Hunger, Nutrition and Health”, food safety is an important area of focus. The World Health Organization cancer agency’s recent advisory on the artificial sweetener, aspartame, and its potential cancer risk, in addition to their new guideline on not using non-sugar sweeteners for body weight control, all together signal a need to revisit how to best study sugar substitutes and other food additives for their potential negative physiological actions as their presence grows in our food supply. This also follows two studies of the NutriNet-Santé population-based cohort that have linked both “artificial sweeteners” as well as “sugar consumption” itself with potentially elevated cancer risks^1,2^. Two more recent studies on aspartame^3^ and erythritol^4^ have brought additional interest in food safety and better product labeling of sweeteners (with most of the field and practitioners recognizing the availability of these sugar substitutes as being needed for those with certain health issues including obesity, diabetes, and heart disease) and flavor enhancers. Of course, sugar itself is contraindicated for these health issues, and should also be studied in all of the paradigms and models discussed here, but like sugar, enhancing the umami taste with additives can also lead to obesity, metabolic syndrome, and brain, e.g., hypothalamic, inflammation which has been described as, “…mediated by nucleotide degradation and uric acid generation…’^5^. The 1969 White House Conference on Food, Nutrition, and Health was organized by Dr. Jean Mayer, for whom the previous institution of food and nutrition science where I was privileged to work was named. Professor Mayer also studied a controversial, umami taste-enhancing food additive, monosodium glutamate (“MSG”), in animal models^6^, and it is still controversial today. Kanarek et al.’s^6^ findings on deficits in caloric restriction and juvenile-onset obesity, along with Mayer’s advocacy and similar reports from other groups, ultimately led to baby food manufacturers suspending MSG use after Congressional Hearings questioned its safety. A conclusion will be reached here that more research and thoughtful product labeling will help the cause of food safety in general, as new consumables and warnings are introduced. In a constantly changing landscape from both new product introductions and new research findings, an open mind is warranted when it comes to supporting or challenging regulation.

There are many critical questions with both the Jones et al.^3^ and Witkowski et al.^4^ studies, but together they provide a powerful impetus for industry and government to come together to help remove stigma over debate of adverse health effects of food additives. This is needed in the meantime until we have better modeling and studying of the distinct human omics, including metabolomics, of food sensitivities within a heterogeneous population offering better stratification of at-risk populations with sensitivities and allergies. This ultimately will lead to easier scrutiny of substances that can trigger adverse health events in certain susceptible individuals who now rely almost totally on truth in labeling, or more accurately, sensitivity- and allergy-guided labeling (since current labeling usually does include names for food components that are truthful, but because of the pervasive use of pseudonyms for many of these additives, it is not obvious or clear enough for easy recognition of any food component that may be undesirable for those with health issues, allergies or sensitivities). All of us are at risk for unrecognized dietary elements that could contribute to potentially serious allergies and sensitivities^7^. Building on the needed use of highly sensitive models and bioassays, and comparisons with carefully run patient studies to determine the actions of dietary components, this Comment hopes to expand the awareness of the significance of studies like these that aim to uncover pathophysiological changes from the consumption of certain food additives. This issue has now been brought to light again from the findings in these new studies on the dietary consumption of artificial sweeteners, aspartame, and erythritol^3,4^, and raises concern again about the safety of additives in our food supply, with potentially adverse health effects that range from neurological to cardiovascular changes. Of considerable concern, the aspartame study reported a transgenerational passage of molecular and physiological changes in the amygdala of a robust animal model, that led to altered neurotransmission and changes in behavior including anxiety in descendants of users^3^. Jones et al conclude their recent study of aspartame’s transgenerational transmission affecting higher function forebrain areas, with the strong suggestion that aspartame’s effect on brain gamma amino butyric acid, “GABA” and glutamate signaling, “…deserves a place on the list of environmental agents such as hormones, insecticides, and drugs of abuse whose adverse effects are not limited to the exposed individuals but manifest in multiple generations of descendants…”^3^. It is noteworthy that this study was done in a rodent model, and therefore before drawing conclusions on human aspartame use, there is a need to see if these findings translate to the human condition. This rodent aspartame study did however attempt to reconcile animal versus human exposure and dosing regimen differences, by having a drinking water dose equivalent to 8–15% of the FDA’s recommended maximum daily intake for humans.

Since aspartame is a derivatized form of the amino acid, aspartic acid, it is also reasonable to consider another widely present food additive for enhancing the savory “umami” taste - another “excitatory amino acid”^8^ and glutamate derivative, monosodium glutamate (“MSG”). It is now ubiquitously present in our food supply, including its increasing presence in synthetic meats because of the need for a savory component. MSG’s potential effects on metabolic and neurologic function^5,6,9,10^ have also been considered as a potential “threat to public health”^11^.

There is debate, triggered in part by the recent publications on aspartame and erythritol, as well as other recently challenged sweeteners and other food additives, within government, industry, and academia regarding the regulation of product labeling of dietary additives, taking into consideration the United States Food and Drug Administration adopting generally regarded as safe, “GRAS”, for many of these products^7,11^. A history of controversy and contentiousness in the description, discussion, and introduction of artificial sweeteners and umami taste enhancers into our food supply has resulted in confusion and to some extent, apathy, amongst scientists, industry, healthcare, and the general public. This may result in part because of a sense of security when it comes to additives that come from natural sources. Erythritol, which the body produces and which also can be found naturally in some foods, is mass-produced for consumption from yeast fermentation. Glucose, sucrose, fucose, erythritol, Stevia, Monk fruit, and other plant-derived sweeteners are different from derivatized compounds like aspartame, whose function may be altered from additive preparation protocols (e.g., adding elements to produce a methyl ester of the aspartic acid/phenylalanine dipeptide, aspartame or “NutraSweet”; or adding sodium to the native chemical structure of glutamate in the case of MSG), are all presumed to be GRAS unless the field finds further evidence of health risk. An argument that these particular additives should not affect one’s physiology any more than the consumption of such substances that the body creates or that occur naturally in certain foods (e.g., erythritol, tomatoes, and mushrooms that contain free glutamate that could have sodium groups added during digestion), needs to be considered in light of introducing boluses of the already derivatized compounds like MSG, in individuals with an extreme sensitivity to the derivatized compound but not to the natural underivatized sources. The two represent completely different processes with different physiological outcomes. In light of the recent publication from the Bhide group^3^, which presented data on altered omics responses from consumption of aspartame, including metabolomics, in studies that unfortunately do not afford direct comparisons of in vitro and in vivo mouse data with such human data, this work supports a conclusion for revisiting the safety of aspartame^12,13^, as well as the other excitatory amino acid derivatives including MSG. This should go beyond the scrutiny of neurological disorders to also have analyses of physiological, biochemical, and metabolic processes that include potential rodent and human cardiometabolic risk^4–6,13^. The Bhide article nonetheless challenged previous attempts at downplaying or questioning potential adverse effects of aspartame, including anxiety disorders in susceptible populations that can be transgenerational transmitted. Just as with this the artificial sweetener, aspartame, another sugar substitute, erythritol, exhibited the potential to contribute to changes in the heart and vascular systems^4^.

The Witkowski et al. study^4^ initially used an untargeted metabolomics approach, combined with a patient study and using blood analysis for quantifying the presence of endogenous erythritol levels, not related to consumption that they did not test in that patient cohort. They used different in vitro and in vivo approaches that don’t necessarily lend themselves to easy comparisons of the different findings from in vitro versus in vivo studies.. They reported that, “…circulating levels of multiple polyols, especially erythritol, was associated with incident (3 years) risk for major adverse cardiovascular events…”, including heart attack and stroke, and their observed effects on thrombosis even though they did not carry out coagulation studies in subjects following erythritol consumption… “, warrant more scrutiny by science, medicine, industry and government since currently, “…The FDA does not require disclosure of erythritol content in food products, making its levels in foods as an additive is hard to track…”^4^. There are apparent methodological and concomitant interpretation shortcomings in the erythritol study because the investigators did not analyze erythritol levels and platelet function after consuming this sweetener, and it is possible that the levels they measured were from endogenous production, potentially from an elevated glucose level and it’s production of erythritol. That said, the author’s goal, as well as that of the current author, is to stimulate more science and clinical studies that are needed for erythritol and all currently used sugar substitutes. Interestingly, MSG has also been a focus of many observational and other epidemiological and experimental studies showing potential adverse effects on both cardiovascular and neurological functions^10–14^, and therefore it has also been considered as a potential “threat to public health”^11^.

The safety of the artificial sweetener, derivatized “excitatory amino acid”^8^ aspartame, has been revisited a great deal over the last several decades^12,13^, ever since Olney and colleagues first introduced the concept that consuming aspartates and glutamates can have profound effects, including negative ones, on our physiology and health^8–10,14^. The new studies (e.g., 3,4) have brought this issue to light once again, this time with the rather surprising altered brain gene expression patterns and other findings on aspartame, relevant to GABA and glutamate transmission in the rodent forebrain. Changes in this GABA and glutamate neurotransmission is associated with anxiety and other affective disorders, and their findings showed that, “…aspartame consumption shifted the excitation-inhibition equilibrium in the amygdala toward excitation…and changes in gene expression were transmitted to male and female offspring in two generations descending from the aspartame-exposed males…”^3^. There certainly may be more than one type of sugar substitute that exhibits potentially adverse physiological effects on both the brain and heart, as presented in another recent study that questioned the toxicity of sucralose and its derivative, sucralose-6 acetate, in a cell culture system using human blood cells, and where DNA strand breaks were produced^15^. And yet another recent study, this time looking at acesulfame-K, a sugar-substitute present in many foods and especially drinks and which is not significantly biodegraded either in our bodies or in the environment as seen in e.g., wastewater analysis^16^, exhibited effects on the state of isolated human blood neutrophils – “homeostasis to priming”^17^. We still do not know all of the health effects of this artificial sweetener, but certainly, there should be freedom of choice to eat foods that contain all of the aforementioned sugar substitutes as well as taste-enhancing food additives like MSG until otherwise regulated. That said, clear and substantive information must be provided on food labels, to afford informed decisions, as well as easy scrutiny and recognition for those who would want to completely avoid them. This requires single names for food additives in such “flavor enhancing” categories, that can be prominently displayed on packaging and thus avoided by those who choose to do so.

It is well accepted that there are foods and nutrients with the ability to affect everything from heart rhythm to brain cognition, plasticity, cancer, and neurodegenerative disease onset and progression^18^. It is possible that such derivatized excitatory amino acids are involved with certain human health conditions involving the nervous or cardiovascular systems including atrial fibrillation. This may not be so surprising in light of MSG often being described by clinicians as having the ability to cause “palpitations” in certain individuals, and atrial fibrillation has also been linked to the combined use of aspartame and monosodium glutamate^14,19,20^. There have been many articles and perspectives written about sugar substitutes versus the use of their “natural” counterparts, in addition to articles about the virtues and risks associated with MSG consumption. The current Comment relied on a nearly coincidental publication of two rather visible research articles on the sugar substitutes aspartame and erythritol to, again, make a call for more truth in labeling and rigorous scientific investigation of these and other sweeteners including the “natural” ones (e.g., sucrose, fructose, and glucose). This should also therefore include flavor enhancers like MSG with similar biochemical and metabolic properties to the other derivatized excitatory amino acid taste-enhancing additives (e.g., aspartame). To date there has been little attention paid to the potential physiological and metabolic effects of derivatized excitatory amino acids (“excitatory” in this case again refers to the free forms of these particular amino acids having excitatory versus inhibitory actions on CNS neurons, with a potential to elicit tissue damaging excitotoxicity^8–10^). This should include the promotion of new and robust multi-omics applications in in vitro and in vivo bioassays of any consumable in question^18^. This recently has been put to the test in a study of a “disease avatar” for use in bioassays, by looking at glial activation and the inflammation-associated hippocampal microenvironment relevant to age-related cognitive decline and Alzheimer’s disease^21^. This work, done in the author’s laboratory, is a proof of principle for this approach and focused on three widely studied, putative anti-inflammatory nutrient agents: curcumin, sulforaphane-rich broccoli sprouts, and epigallocatechin-3-gallate (EGCG) from green tea, individually and in combination. It was found that, together, they had the ability to attenuate the tissue-increased inflammatory level of chemically-stressed hippocampal neurons and microglia in cell culture^21^. Such work^18,21^ represents a new approach for studying discrete effects of a combination phytonutrient for positively affecting human physiology and health, and is applicable to bioassays for any food or nutrient component.

In all, for the author, this story is respective and it was originally intended to be a firsthand essay in part inspired by the publication of the Bhide and Witkowski groups’ articles. A pragmatic self-understanding of the etiology of such additive sensitivities, from experiential in addition to the perspective gained from physiology, molecular medicine, and nutrition science of neurological and cardiovascular risk including arrhythmias, consumption of aspartame with subsequent unintended exposures to MSG led to attempts at preventing and mitigating, in my case, notable consequences. Carrying a list, e.g., of almost twenty commonly used names for the umami flavor enhancer and its sources, and handing it to willing food servers for their conferring with the kitchen about any foods that might contain MSG or any of its pseudonym sources, especially from prepackaged vendors rather than made in that kitchen, ultimately proved to be inadequate. With such attempted vigilance, the burden of too many names for MSG to afford scrutinization of every prepared food item label in a restaurant’s kitchen ultimately led to, at best, confusion and avoidance, or in the worst case scenario from casting fate to the wind, countless cardiac events over the years. In many ways, the derivatized glutamate, MSG, is not so different from the aspartic acid derivative, aspartame, with many previous studies including some that associated the use of aspartame and MSG with atrial fibrillation in certain people^19^. Focusing on the two articles about aspartame and erythritol^3,4^ afforded an opportunity to bring personal experience and knowledge into the discussion of the potential that there are others, like me, who might also have just the perfect omics storm, especially genomics, and metabolomics, that can contribute to potentially extreme sensitivities to such derivatized excitatory amino acids. The sugar substitutes, as well as their natural food targets, MSG and other additives, and for that matter, any consumable, can be modeled and robustly studied in the laboratory as well as their in-human use through carefully designed clinical trials that include extensive multi-omics analyses, both from the patient as well as from their experimental “avatar”^18,21^.

Thus, there is a need to encourage both further research and especially dialogue amongst investigators about the need for a better understanding of the physiology and potential toxicity of flavor enhancers for particular at-risk individuals. This holds promise to help those with extreme sensitivities to even small quantities of a particular additive, to not have to deal with many confusing names for it that they may not tolerate well. There is a need for utilizing more sensitive bioassays, including controls and human subjects with health issues and food sensitivities or allergies, in a dietary-risk “avatar” model that possesses personalized risk and disease elements such as immune and relevant at-risk tissue cells, for deep interrogation that could include machine learning of the cell and molecular biology of therapeutic and potentially contraindicated foods and nutrients^18^.

The umami taste enhancer, MSG, including any of its pseudonym sources e.g., hydrolyzed vegetable protein(s) of different vegetable origins, autolyzed yeast extract, and Torula yeast, increases appetite via stimulating the savory flavor and has been implicated in metabolic syndrome, as well as potentially contributing to cardiac and central nervous system changes (5, 6, 9, 10, 11, 14). MSG is believed to stimulate the release of glucocorticoids and catecholamines from the adrenal glands^22,23^, that can affect the heart directly or through other systemic metabolic and inflammatory actions that can lead to changes in heart rate and/or rhythm. Similar changes in brain electrophysiology via altering glutamate and GABA neurotransmission could affect numerous connectional and molecular pathways leading to cardiovascular, behavioral, and cognitive changes. Many synthetic derivatives, along with increasing numbers of other names for sources of MSG, are appearing on food labels that make surveillance and thus avoidance difficult. It is noteworthy that previous work has suggested the possibility of cell and tissue, including both heart and brain, excitotoxicity resulting from consumption of so-called excitatory amino acids and their derivatives^8^. These compounds have been reported to reach sites in the central nervous system possessing a weak blood–brain barrier, including circumventricular organ access to the chief autonomic and neuroendocrine center of the brain, the hypothalamus^9,10,24^. Excitotoxicity also has been discussed in relation to gliomagenesis^25^ and possibly other pathological conditions^26^.

Sugar substitutes, the aspartates and glutamates, or any other additive or food component in question, can be further examined in future epidemiological studies, and despite the nutritional science field acknowledging that well-controlled human and behavioral studies are difficult, the precise effects of dietary components on human physiology and behavior can be determined. As has been applied in the Jones et al study of an artificial sweetener like aspartame^3^, or as attempted in the erythritol study^4^, any consumable in question could be assayed both ex vivo and in highly controlled and carefully executed human behavioral paradigms, e.g., as shown by Brickman et al looking at cocoa flavonoid’s role in supporting human memory function^27^. It is notable that the field of oncology is paying close attention to such approaches for also studying food as medicine, e.g., supporting cancer therapies, and in silico and other screens are beginning to uncover natural product sources of oncogenic-network-mediating drugs. For example, a study of WWP1- of the human tumor suppressor gene, PTEN, has uncovered potent inhibitors of this oncogenic axis, derived from cruciferous vegetables in a mouse avatar model^28^. There is a need for reinforcing findings on dietary component contributions to normal and pathophysiological functions, via comparison with in vitro and in vivo animal modeling experiments of the same dietary components. Robust in vitro cell and tissue preparation assays, along with in vivo animal models (transgene, knockout, and at-risk cell xenografts) that focus on gene expression patterns, cell biology, physiology, and behavior that may be affected by any food component in question, can help establish generalized effects of foods and food products on human central nervous system, heart as well as other tissue and organ function^18^.

It is reasonable to suggest that following a similar protocol as presented for the screening of neuroactive, regeneration-supporting, or nutrient components^18^, food additives and other dietary elements can be directly assayed in such models for their potential contributions to sensitivities and counterproductive health effects in susceptible individuals. This can employ state-of-the-art methods including omics, cell, molecular and systems biology, where together, “…Any dietary intervention, whether for purposes of diagnosis or management of a food allergy or intolerance, should be adapted to the individual’s dietary habits… [and sensitivities to additive ‘food chemicals’]… ”^7^, and one’s individual omics and nutritional requirements, that together can help prevention and mitigation of related health challenges. At the same time, these approaches offer the potential for deep characterization and stratification of populations of people at risk for diet-associated cardiac arrhythmias, and other heart and brain disorders. These patients may be amenable to diet, lifestyle, and behavioral modifications as an adjunctive therapeutic approach to be used along with standard-of-care medicine or emerging treatments for any health malady they are also having to deal with.

With more epidemiological and modeling studies providing data on food and food additive sensitivities, it is hoped that both industry and government will respond with better documentation of potential non-IgE allergies and sensitivities. This should not affect access from the population who wants and may not have the risk for the dietary contribution in question, but rather it would allow at-risk populations the opportunity for easier scrutiny and avoidance. Ultimately, people who desire to keep particular sugar substitutes and flavor enhancers in their diet, irrespective of possible food sensitivities that could put them at risk for health challenges, should have the right to purchase and consume these products. As pointed out by Witkowski et al.,^4^, their findings regarding erythritol, “… highlight the need to establish reporting requirements, safety profiles and margins of daily intake amounts given that broad consumption continues to increase. Public policy decisions need to be evidence-based and better informed…” But with regard to regulation, it is important that we remain open to emerging findings that could inform revisions or the addition of new guidelines. It is clear that the area of nutrition science that focuses on sweeteners and flavor enhancers is constantly being defined and redefined. There is a need for human clinical studies that, in addition to strategically complementing laboratory and modeling studies, examine the effects of any consumable that is purported to be neither safe nor healthy in a longer time period and randomized controlled trials, that include a greater number of subjects with food sensitivities, especially to that consumable. In addition to the need for more science, and the need to increase our knowledge of the physiological actions of different sugar substitutes and taste-enhancing additives in our diet, it behooves us to have truth in package labeling, with simple and clear names for compounds and components present in our foods, since we all possess allergies and sensitivities^7^ that from ongoing advances in bioassays and data science should afford us easier scrutiny of any food component, and informed decision-making on its use.

## Reporting summary

Further information on research design is available in the Nature Portfolio Reporting Summary linked to this article.

### Supplementary information

Reporting summary

## Data Availability

All data discussed in this Comment are fully available in the main text and cited articles available in PubMed.

## References

[1] Debras C (2022). Artificial sweeteners and cancer risk: results from the NutriNet-Santé population-based cohort study. PLOS Med..

[2] Debras C (2020). Total and added sugar intakes, sugar types and cancer risk: resuts from the NutriNet-Santé prospective cohort. Am. J. Clin. Nutr..

[3] Jones SK (2022). Transgenerational transmission of aspartame-induced anxiety and changes in glutamate-GABA signaling and gene expression in the amygdala. Proc. Natl Acad. Sci. USA.

[4] Witkowski, M. et al. The artificial sweetener erythritol and cardiovascular event risk. *Nat. Med*. 10.1038/s41591-023-02223-9 (2023). 10.1038/s41591-023-02223-9 PMC10334259 36849732

[5] Andres-Hernando A (2021). Umami-induced obesity and metabolic syndrome is mediated by nucleotide degradation and uric acid generation. Nat. Metab..

[6] Kanarek R, Meyers J, Meade RG, Mayer J (1979). Juvenile-onset obesity and deficits in caloric regulation in MSG-treated rats. Pharmacol. Biochem. Behav..

[7] Skypala, I. J., Williams, M., Reeve, L., Meyer, R. & Venter, V. Sensitivity to food additives, vasoactive amines and salicylates: a review of the evidence. *Clin. Transl. Allergy*10.1186/s13601-015-0078-3 (2015). 10.1186/s13601-015-0078-3 PMC4604636 26468368

[8] Olney JW (1994). Excitotoxicity in foods. NeuroToxicology.

[9] Olney JW (1969). Brain lesions, obesity and other disturbances in mice treated with monosodium glutamate. Science.

[10] Olney JW (1971). Monosodium glutamate effects. Science..

[11] Niaz K, Zaplatic J (2018). Extensive use of monosodium glutamate: a threat to public health?. EXCLI J..

[12] Choudhary AK, Pretorius E (2017). Revisiting the safety of aspartame. Nutr. Rev..

[13] Fowler SPG (2016). Low calorie sweetener use and energy balance: Results from experimental studies in animals, and large scale prospective studies in humans. Physiol. Behav..

[14] Zanfirescu A (2019). A review of the alleged health hazards of monosodium glutamate. Compr. Rev. Food Sci. Food Saf..

[15] Schiffman, S., Scholl, E. H., Furey, T. S. & Nagle, H. T. Toxicological and pharmokinetic properties of sucralose-6-acetate and its parent sucralose: in vitro screening assays. *J. Toxicol. Environ. Health Part B*10.1080/10937404.2023.2213903 (2023). 10.1080/10937404.2023.2213903 37246822

[16] Belton K, Schaefer E, Guiney PD (2020). A review of the environmental fate and effects of acesulfame-potassium. Integr. Environ. Assess. Manag..

[17] Skurk T (2023). Sweetener system intervention shifted neutrophils from homeostasis to priming. Nutrients.

[18] Steindler DA, Reynolds BA (2017). Perspective: neuroregenerative nutrition. Adv. Nutr..

[19] Burkhart GC (2009). Lone atrial fibrillation precipitated by monosodium glutamate and aspartame. Int. J. Cardiol..

[20] O’Keefe EL, Sturgess JE, O’Keefe ,JH, Gupta S, Lavie CJ (2021). Prevention and treatment of atrial fibrillation via risk factor modification. Am. J. Cardiol..

[21] Fisher DR (2022). Phytochemical combination is more effective than individual components in reducing stress signaling in rat hippocampal neurons and microglia in vitro. Int. J. Mol. Sci..

[22] Perello M, Gaillard RC, Chisari A, Spinedi E (2003). Adrenal enucleation in MSG-damaged hyperleptinemic male rats transiently restores adrenal sensitivity to leptin. Neuroendorcrinology.

[23] Morrison JFB (2007). Sensory and autonomic changes in the monosodium glutamate-treated rat: A model of type II diabetes. Exp. Physiol..

[24] Price MT, Olney JW, Lowry OH, Buchsbaum S (1981). Uptake of glutamate and aspartate by the circumventricular organs but not other regions of brain. J. Neurochem..

[25] Rothstein JD, Brem H (2001). Excitatoxic destruction facilitates brain tumor growth. Nat. Med..

[26] Cavalheiro EA, Olney JW (2001). Glutamate antagonists: deadly liaisons with cancer. Proc. Natl Acad. Sci. USA.

[27] Brickman A (2014). Enhancing dentate gyrus function with dietary flavanols improves cognition in older adults. Nat. Neurosci..

[28] Lee Y-R (2019). Reactivation of PTEN tumor suppressor for cancer treatment through inhibition of a Myc-WWP1inhibitory pathway. Science.

